# The Intricate Relationship between Type 2 Diabetes Mellitus (T2DM), Insulin Resistance (IR), and Nonalcoholic Fatty Liver Disease (NAFLD)

**DOI:** 10.1155/2020/3920196

**Published:** 2020-07-31

**Authors:** Daniela Maria Tanase, Evelina Maria Gosav, Claudia Florida Costea, Manuela Ciocoiu, Cristina Mihaela Lacatusu, Minela Aida Maranduca, Anca Ouatu, Mariana Floria

**Affiliations:** ^1^Department of Internal Medicine, “Grigore T. Popa” University of Medicine and Pharmacy, Iasi, Romania; ^2^Internal Medicine Clinic, “Sf. Spiridon” County Clinical Emergency Hospital, Iasi, Romania; ^3^Department of Ophthalmology, “Grigore T. Popa” University of Medicine and Pharmacy, Romania; ^4^2nd Ophthalmology Clinic, “Prof. Dr. Nicolae Oblu” Emergency Clinical Hospital, Iasi, Romania; ^5^Department of Pathophysiology, Faculty of Medicine, “Grigore T. Popa” University of Medicine and Pharmacy, Iasi, Romania; ^6^Unit of Diabetes, Nutrition and Metabolic Diseases, “Grigore T. Popa” University of Medicine and Pharmacy, Iasi, Romania; ^7^Clinical Center of Diabetes, Nutrition and Metabolic Diseases, “Sf. Spiridon” County Clinical Emergency Hospital, Iasi, Romania; ^8^Department of Physiology, “Grigore T. Popa” University of Medicine and Pharmacy, Iasi, Romania; ^9^Internal Medicine Clinic, Emergency Military Clinical Hospital, Iasi, Romania

## Abstract

Nonalcoholic fatty liver disease (NAFLD) and type 2 diabetes mellitus (T2DM) remain as one of the most global problematic metabolic diseases with rapidly increasing prevalence and incidence. Epidemiological studies noted that T2DM patients have by two-fold increase to develop NAFLD, and vice versa. This complex and intricate association is supported and mediated by insulin resistance (IR). In this review, we discuss the NAFLD immunopathogenesis, connection with IR and T2DM, the role of screening and noninvasive tools, and mostly the impact of the current antidiabetic drugs on steatosis liver and new potential therapeutic targets.

## 1. Introduction

The liver is one of the main houses that control the metabolic homeostasis. Metabolic diseases such as obesity, IR, T2DM, dyslipidaemia, and NAFLD are connected through molecular-biochemical, and complex immune mechanism [1, 2]. Both diabetes and NAFLD are chronic diseases that usually portray nonalarming changes that can lead to disability and many other metabolic complications. They are all independently mortality and morbidity risk promoters, and overall global financial consumer disorders [3–5].

Currently, NAFLD remains one of the most frequent liver diseases, affecting up to 25% of the general adult population [6–8], and with reported increased incidence in children [9]. Soon it may become the most common indication for liver transplant [10]. This multifactorial condition can derive from an unhealthy lifestyle, obesity, dyslipidaemia, type2 diabetes mellitus, and/or other metabolic syndromes [11, 12]. It is characterized by a wide spectrum of liver diseases that vary from simple fat accumulation (benign steatosis), to inflammation (nonalcoholic steatohepatitis (NASH)), fibrosis, cirrhosis, liver failure, and finally to hepatocellular carcinoma (HCC), in the absence of excessive alcohol consumption, medications, or viral aetiology [13–16]. Researchers found that individuals with diagnosed NAFLD have a two-fold increased risk of T2DM [17], and higher risk to develop oncologic [18], cardiovascular [19, 20], and renal disease [21] especially when is associated with T2DM [22].

By now, TD2M is reported to affect 1 in 11 adults and up to 463 million people worldwide [23]. Since the 90s, there is an increased incidence and prevalence of prediabetes and T2DM among the paediatric population which is linked to a high-fat diet, sedentarism, obesity, and liver-related diseases [24, 25]. As T2DM is defined by high serum glucose levels, IR, and damaged islet cell function, it is possible that patients with NAFLD have a higher risk of developing diabetes as they usually express abnormal glucose metabolism [26]. Interestingly, recent evidence shows that T2DM is an independent risk factor for NAFLD [27], women with a history of Gestational Diabetes Mellitus (GDM) have a higher risk of NAFLD, and vice versa [28, 29], and that hepatic steatosis resolution can prevent T2DM onsets [30, 31].

Over the years, substantial efforts were made in order to elucidate the immunopathogenic mechanism behind NAFLD and its connections with T2DM. Even if they are still insufficiently known data, IR seems to be one of the key events that appear in both disorders [32, 33]. The interplay between T2DM, NAFLD, and IR could be considered a two-way street. It is difficult to establish if IR is the cause or the consequence of NAFLD and T2DM, and what is the full relationship with other metabolic syndromes [34, 35]. Nonetheless, it is very important to understand as much as possible this codependency relationship.

In this review, we aim at describing the immunopathogenesis behind NAFLD, how IR is the hallmark that coexist in booth diseases, how we can prevent and assert hepatic changes through noninvasive methods, what are the best therapeutic approaches in T2DM subjects that have NAFLD, and the new desirable possible therapeutic options, and foremost we hope to raise awareness among clinicians about how we should look beyond one disease and the importance of screening for better prevention, management, and outcomes.

## 2. Immunopathogenesis

The link between T2DM and NAFLD can be described by a spectrum of metabolic changes represented by IR, defective hepatic lipidic profile, and triglyceride (TG) metabolism which lead to fat accumulation, immune responses, and/or subsequently hyperinsulinemia determined by the *β*-cell dysfunction in T2DM [36]. Normally, there is a balanced scale between lipid uptake (free fatty acids (FFAs) or “de novo lipogenesis” (DNL), and esterification) and lipid disposal (metabolism or *β*-oxidation, and elimination as very-low-density lipoproteins (VLDL)). In NAFLD, VLDL removal cannot keep up with the increased rate of TG uptake and intrahepatic production [37]. Thus, NAFLD immunopathogenesis can be described by two hypotheses. One that includes increased intake of dietary fats that lead to free fatty acids (FFAs) surplus, increased DNL, and decreased hepatic TG excretion, and one that encompasses oxidative stress, lipid peroxidation, mitochondrial dysfunction, and release of inflammatory mediators [29, 38, 39].

### 2.1. Lipotoxicity

To maintain strict control of the hepatic lipid homeostasis, compound interactions are made between hormones, nuclear receptors, and transcription factors [40]. As known, carbohydrate excess contributes to steatosis via DNL that produces lipogenic molecules such as acetyl-CoA carboxylase (ACC), fatty acid synthesis (FAS), and stearoyl CoA-desaturase-1 (SCD-1). DNL plays a very substantial role in the development of NAFLD and is characterized by a series of enzymatic transformations. First, glucose is converted to acetyl CoA by glycolysis and pyruvate oxidation. Acetyl-CoA is then converted to malonyl-CoA by ACC. FAS catalyse the formation of palmitic acid from malonyl-CoA and acetyl-CoA. Palmitic acid is then desaturated by long-chain fatty acid elongase 6 and SCD1 to generate saturated fatty monoacids, which are the main constituents of triglyceride fatty acids. Glycerol-3-phosphate acyltransferase (GPAT) then catalyses the esterification of glycerol-3-phosphate from glycolysis with newly synthesized fatty acid to phosphatidic acids. The phosphatidic acids are then processed into diacylglycerols (DAG) by lipin, followed by the formation of triglycerides by acyl-CoA: diacylglycerol acyltransferase [41–43]. Glucose and insulin promote lipogenesis through activation of the carbohydrate response element-binding protein (ChREBP) and the sterol regulating element-binding protein 1c (SREBP1c) [38, 44]. Fructose increases the expression and induction of CD36 lipogenic pathways, including uniquely regulation of ChREBP and SREBP1c, increased steatosis, and reduced hepatic insulin signalling [45, 46]. SREBPs are the most skilled regulators of lipid uptake and cholesterol biosynthesis, and can induce liver steatosis by enhancing TG expression. Deletion of SREBP-1a promotes toll-like receptor-4 (TLR4) stimulation, increased inflammatory gene expression via reprogramming the fatty acid production [47], systemic IR, and reduction of the glucose transport [48]. Transcriptional regulation of DNL is primarily orchestrated by SREBP1c. This process connects DNL to cholesterol metabolism and indirectly to IR, mainly because SREBP1c can enhance the generation of harmful lipid molecules such as DAG and ceramides which further enhance IR, resulting in a positive feedback loop, in which hepatic DNL help IR and IR stimulate hepatic DNL [49]. Ceramides are an additional class that combines cellular toxicity with proinflammatory actions. Ceramides contribute to inflammation through interaction with TNF*α*, are involved in oxidative stress, and cell death [50]. They damage the mitochondria and the endoplasmic reticulum (ER) function, elicit oxidative stress, promote apoptosis, and histologic dysmorphic liver lesions like ballooning and Mallory-Denk bodies [45, 51]. In addition, lipid overload in the pancreatic *β*-cells impels insulin secretion and changes the expression of peroxisome proliferator-activated receptor- (PPAR-) *α*, glucokinase, glucose transporter 2, preproinsulin, and of pancreatic duodenal homeobox, which enhance IR as a result of apoptosis [52]. The PPAR-*γ* is a transcriptional regulator of adipose metabolism that binds to SREBPs. Experimental data shows that in obese models PPAR-*γ* and SREBP1 expression are elevated [53] and that SREBP-1c/PPAR*α* ratio can be used as an index of hepatic steatosis [54].

### 2.2. Oxidative Stress

As seen, lipid excess leads to fat storage accumulation, abnormal lipid peroxidation, the release of the proinflammatory cytokine, high reactive oxygen species (ROS), and reactive nitrogen species (RNS). Lipid peroxidation then promotes stellate cell proliferation, which contributes to fibrogenesis. The nuclear erythroid 2-related factor 2/antioxidant response element (NRF2/ARE) pathway which modulates the antioxidant effect of ROS and RNS is flawed in subjects with obesity and IR [55]. ROS induce the release of cytokines from hepatocytes, trigger TLR-4 synthesis, and promotes inflammatory liver macrophage activation [56].

### 2.3. Hepatic Cell Activation

In major metabolic diseases, the onset is characterized by alteration of peripheral macrophage number and functional phenotype, especially in the hepatic and obese tissue [57]. In increased adipose tissue, researchers found high levels of macrophages, mainly resident adipose tissue macrophages (ATMs), which were detected in clusters named crown-like structures (CLS) [58, 59]. These ATMs are fundamental for tissue homeostasis, are involved in tissue remodelling, clearance of cellular debris, inflammation, and fibrosis [60–62]. In human lean adipose tissue, ATMs are represented by CD14+/CD16− and express markers like CD11c+ and CD206 that are associated with IR [63]. The elements that activate ATMs into metabolically activated macrophage are the lipoproteins, FFAs, glucose, and insulin. Liver macrophages (LM) are transformed from an anti-inflammatory (M2) to a proinflammatory cell function (M1) [64, 65]. M1 enhances the production of chemokines such as chemokine ligand 2 (CCL2) that induces the synthesis of tumor necrosis factor-alpha (TNF-*α*), IL-1 *β*, and interleukin-6 (IL-6) which can alter the insulin sensitivity in the adipose tissue [66, 67]. Importantly, TNF-*α* activates two major proinflammatory signaling pathways: the c-Jun N terminal kinase (JNK) a mitogen-activated protein kinase family member and the nuclear-kappa B (NF-*κ*B) pathway, both linked to IR and NAFLD. Under normal conditions, NF-*κ*B is sequestered in the cytoplasm and binds to the inhibitor of kappa B (I*κ*B) proteins, which then inhibit the nuclear localization of NF-*κ*B. The NF-*κ*B kinase of nuclear factor kappa-B kinase (IKK-*β*) inhibitor plays an important role in the activation of NF-*κ*B. It seems that the deletion of IKK-*β* improves glucose tolerance and insulin sensitivity and suppresses the NF-*κ*B pathway which can limit the lipogenesis and inflammation processes [64, 68]. Also, TNF-*α* reduces AMP-activated protein kinase (AMPK) activity which has a role in NAFLD development [69]. The JNK pathways constant activation is maintained by stimuli-like oxidative stress or various drug via a feedback loop mechanism. Subsequently, JNK phosphorylates insulin receptor substrate (IRS) which instigates the inhibition of the insulin signaling [70, 71].

Kupffer cells (KCs) are the most flourishing population of resident macrophages that inhabit the liver. Among the immune homeostasis regulation, KCs coordinate the metabolism of bilirubin, cholesterol, iron [72, 73], and can recruit neutrophils and natural killer T cells (NKT-cells) into the liver [74]. This recruitment is modulated by chemotactic factors, such as monocyte chemotactic protein-1 (MCP-1). MCP-1 production is initiated by hepatocytes during simple steatosis and is supported by the infiltrating macrophages. Blocking or absence of MCP-1 or C-C Motif Chemokine Receptor2 (CCR2) the receptor for MCP-1 reduces the influx of monocytes and macrophages into the liver, effectively stopping the development and evolution of inflammation and fibrosis [75–77]. Studies show that CCR2+ macrophage is a pioneer in the hepatic monocyte uptake; it can induce lipolysis through regulation of epinephrine and norepinephrine levels and promote liver injury [78]. In T2DM-NAFLD subjects, authors found increased levels of Fatty acid-binding protein 1 (FABP1), a protein that facilitates the storage of FFAs and urges liver damage [79, 80]. Some suggested that this protein may be used as a biomarker to detect liver injury [81]. Another key protein is the Fatty acid transport protein 1 (FATP1) which along with FATP4/5 contributes to the inflammatory macrophages function. Deletion of FATP1 causes glucose intolerance, whereas inhibition/deletion of FATP4/FATP5 has beneficial metabolic effects [82]. An indispensable component of the amino acid metabolism that provides macrophage activation and polarization is glutamine. Glutamine attenuates inflammasome activation, macrophage cell death and has overall beneficial effects. In M1 macrophages, glutamine intensify their proinflammatory effects via succinate in response to LPS, enhances lipotoxicity which promotes inflammation in adipose tissue and trigger IR. Subjects with diabetes or obesity usually have decreased glutamine and increased succinate concentrations [83, 84]. Notably, oral supplementation with glutamine ameliorated diet-induced NASH progression in C57BL/6J mice models [85]. This vicious loop of inflammatory events that are connected and influenced by IR may provide new tools for detection, or new therapeutic targets that can stop the onset and progression of NAFLD.

### 2.4. Adipokines

Obesity is a major risk factor for diseases like T2DM, hyperlipidemia, and NAFLD. This metabolic disease emerges from an imbalance in energy input, energy consumption, and fat accumulation [86]. Adipose tissue is a well endocrine organ that secretes hormones and cytokines known as adipokines. The development of IR in NAFLD is also likely related to the imbalance between proinsulin (adiponectin, leptin) and anti-insulin (i.e., TNF*α*) cytokines [87–89]. Adiponectin is a specific secretory adipokine that regulates of fatty acid oxidation (FAO), inhibits the accumulation of FFAs, maintains the glucose homeostasis throughout the body, and the sensitivity to hepatic insulin. Hypoadiponectinemia affects the metabolism of fatty acids and promotes a chronic state of inflammation in the liver [90]. On the other hand, leptin can impel the hepatic stellate cell activation and liver fibrosis, control the energy balance, and suppress appetite [91]. Increased levels of leptin were found in subjects with increased body fat and cardiometabolic disorders [92]. Authors noted in a cross-sectional study that NAFLD patients have lower adiponectin levels, higher serum leptin levels, and higher leptin-to-adiponectin (L/A) ratio [93]. Adiponectin and leptin can independently predict the onset of NAFLD that is why they may be taken into consideration as potential predictive biomarkers for NAFLD [94]. Recently, researchers found that novel adipokine Gremlin 1 can antagonize insulin signaling, is positively correlated with the percentage of body fat and IR in T2DM and NAFLD/NASH subjects, and also could represent a potential biomarker or therapeutic target [95].

### 2.5. Gut Microbiota

Another theory suggest that gut microbiome alteration and dietary habits are another mechanism that induce and maintain T2DM and/or NAFLD [96, 97]. Indeed, data shows that gut dysbiosis enhances bacteria production that can regulate KCs inflammatory activation, promotes short-chain fatty acids (SCFAs) production, changes the enterohepatic circulation of bile acid, and can lead to inflammation and finally hepatic steatosis [98, 99]. Gut-derived metabolites such as tryptophan modulate inflammatory responses in the macrophages and in hepatocytes [100]. Inflammasome key protein complexes when activated induce cell apoptosis and proinflammatory cells release and are essential in host defences mechanism. NLRP3 inflammasome contributes through gut microbiota to control the NAFLD/obesity progression via overproduction of leptin, downregulation of adiponectin generation, and promotion of fibrosis [101]. Circulating microbiota-derived metabolites could be used for NAFLD diagnosis [102].

### 2.6. Insulin Resistance

**Figure 1:**
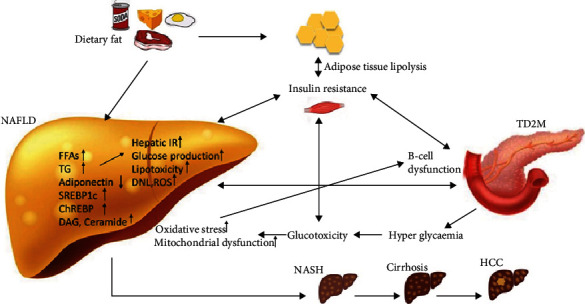
NAFLD, IR, and T2DM complex immunopathogenesis. T2DM: type 2 diabetes mellitus; NAFLD: nonalcoholic fatty liver disease; NASH: nonalcoholic steatohepatitis; HCC: hepatocellular carcinoma; IR: insulin resistance; FFAs: free fatty acids; TG: triglyceride; ChREBP: carbohydrate response element-binding protein; SREBP1c: sterol regulating element-binding protein 1c; DAG: diacylglycerols; ROS: high reactive oxygen species;

Insulin is an anabolic hormone that can mediate the fluid homeostasis, ionic transport, storage of TG in the adipose tissue, can promote esterification and storage of fatty acids in lipid droplets, and can inhibit the lipolysis. Under normal conditions, the pancreatic *β*-cells are secreting insulin after a meal or after hormone release (i.e., catecholamine, glucagon). Insulin suppresses the production of hepatic glucose and stimulates peripheral glucose uptake, while hormones such as glucagon-like peptide-1(GLP-1) stimulates gluconeogenesis, glycogenolysis, and hepatic glucose production. Insulin mediates the glucose metabolism not only by promoting glucose uptake by the adipose tissue and by the hepatic tissue but also by suppressing the hepatic glucose production [103]. Hepatic insulin clearance is dampened in T2DM subjects and is correlated with the metabolic syndrome severity. In fact, insulin exhibits both anti-inflammatory and proinflammatory properties [104]. The term “insulin resistance” is generally used to describe insulin-mediated glucose uptake in the skeletal muscle. IR is defined by suboptimal cellular response to physiological levels of insulin in diverse tissues. This is the pioneer that in critical conditions, increases glycolysis, and release of FFAS for peripheral needs, as most of the glucose, is directed to the brain [40, 105]. Subsequently, hyperinsulinemia results from the beta cells effort to overcome IR by enhancing insulin release. High caloric intake damages the insulin receptor signaling resulting in a flawed suppression of FFAs release from the adipose cells and also flawed nitric oxide (NO) release [106]. Hence, IR and inflammation form a vicious circle, each condition promoting the other and accelerating the development of NAFLD and other metabolic disorders in the presence of lipotoxicity [107]. In both obese and lean subjects, high IR was found to be the most significant predictive factor for NAFLD [108], and research showed that serum insulin levels are firmly associated with ballooning and hepatic lobular inflammation [109]. The intricate relationship between IR, NAFLD, and T2DM is based on a vicious circle. Obesity induced by a high-fat diet is the main precursors that trigger the lipotoxicity and the glucotoxicity pathways which are both mediated by insulin through IR (Figure 1.).

## 3. Screening and Assessment

Steatosis liver is defined when liver fat exceeds 5% of hepatocytes in absence of other secondary causes for lipid hepatic accumulation or by >5.6% of proton density fat fraction measured by MRI/spectroscopy [110]. Even if usually NAFLD is detected by chance through noninvasive imaging, the gold standard for NAFLD diagnosis remains the liver biopsy. However, this procedure is invasive has many serious side effects and is expensive. Thus, it is imperative to develop new systems and guidelines that include, screening, serological, and noninvasive imaging methods to help prevent and diagnose NAFLD in TD2M patients. Recently Bertot et al. [111] reported that noninvasive scoring systems are less accurate at liver outcome prediction in individuals with NAFLD and diabetes. Further data is needed so that we can underline a robust and firm conclusion regarding the practical use of scoring systems. As seen, individuals with T2DM have an increased risk of developing moderate-severe liver damage and have a higher chance to develop HCC [112]. In patients with prediabetes or T2DM serial liver biopsies revealed progressive fibrosis [113]. Scientific research found that Liver Index (FLI) and GGT correlate with peripheral IR and the risk of prediabetes, diabetes, and hypertension development [114, 115]. IR remains one of the key pathogenic tools that onset and maintains the progression of NAFLD to NASH. Homeostasis Model Assessment of Insulin Resistance (HOMA-IR) and liver stiffness measurement (LSM) using acoustic radiation force impulse (ARFI) elastography correlated with the liver fibrosis grade in obese-NAFLD patients [116]. These results can be attributed to the fact that obese subjects have higher peripheral insulin and lower hepatic insulin clearance. Histological alterations are positively correlated with parameters of IR and authors suggest that in the near future 2-hour oral glucose tolerance test (OGTT) may be used to assess NAFLD severity [117]. Along with the OGTT, authors recommend that Impaired Fasting Glycemia (IFG) should be used in the young population for screening at-risk of metabolic syndrome development [118]. Also, the Hepatic steatosis index (HSI) may be a useful tool in the primary screening of NAFLD [119]. So far, a proper screening for NAFLD in TD2M patients is currently unavailable because of the limits of the noninvasive diagnostic tools and the lack of therapeutic options. Nonetheless, every T2DM subject has one or more risk factors to develop liver damage and should be periodically checked [120]. The newly ADA (American Diabetes Association) guidelines updates recommend that individuals with prediabetes or T2DM that have elevated hepatic enzymes or steatosis liver should be investigated for fibrosis/NASH through noninvasive techniques [121].

The most known techniques approved by the European Association for the Study of Diabetes (EASD) [122] include NAFLD liver fat score (NLFS) and the fatty liver index (FLI) for NAFLD diagnosis [123], and fibrosis-4 index (FIB-4) and NAFLD fibrosis score (NFS) for fibrosis assessment [124]. Also, known the SteatoTest, NashTest, ActiTest, FibroTest, and FibroScan are handy tools in quantifying liver impairment [125]. FibroScan® is a reliable instrument in detecting and staging fibrosis in NAFLD/NASH that can also identify macrovascular and microvascular complications of diabetes [126, 127]. Experts propose as the gold standard for detection and grading of the hepatic steatosis, using the intrahepatic TG measurement with the magnetic resonance imaging derived proton density fat fraction (MRI-PDFF) [128, 129]. Subjects with intermediate or high risk of severe fibrosis may benefit from FibroMeter that performs better than the simple FIB4 and NFS tests, and can measure the hepatic extracellular matrix components [130]. Also, Ampuero et al. [131] developed and validated recently the Hepamet fibrosis scoring system, a noninvasive scoring test with 97.2% specificity and 74% sensitivity that uses clinical and laboratory information from NAFLD subjects and can identify fibrosis stage with better accuracy than NFS and FIB-4.

## 4. Therapeutic Options

Since NAFLD has a complex immunopathogenesis, is dependable by numerous exogenous and endogenous factors, and has tight associations with other metabolic disease, a few therapeutic strategies are available. Efforts are currently in progress to find new promising treatment options to combat NAFLD [122]. In lack of a concrete pharmacotherapy, the current guidelines recommendation for NAFLD mainly emphasize revisions of lifestyle [108, 132].

### 4.1. Dietary and Lifestyle Revisions

We know that genetics, heritability, and gender type are major factors that raise the susceptibility to develop T2DM, IR, NAFLD, obesity, and/or other metabolic syndromes [133, 134]. While these cannot be changed, factors such as the circadian rhythm that interestingly promotes metabolic disruptions [135, 136], lifestyle (exercise, weight loss), and dietary changes can improve clinical and paraclinical outcomes of NAFLD and T2DM on long term [137, 138]. More than that, continuous exposure to environmental factors like endocrine-disrupting chemicals such as the ubiquitous phthalates and heavy metals adversely affect human health. These synthetic phthalate esters are found anywhere around us from air, to industrial products, and industrial food [139]. They act like hormones and interfere with different receptors such as PPAR-*α*, as well as androgen receptors (AR), thyroid hormone receptors (TR*α*, TR*β*) which interrupt the normal lipid and glucose homeostasis. There is sufficient evidence that links metabolic disorders development through phthalates exposure [140]. This shows that not only personal habits are important but also the overall living condition. Recently, The Diabetes Remission Clinical Trial (DiRECT) noted that weight loss in TDM patients led to liver fat loss and recovery of the *β*-cell function [141]. Also, in a recent systematic review and meta-analysis, authors observed that caloric deficient diet and periodic exercise ameliorated hepatic functions [142]. Low intake of fibres, vitamins, and mineral nutrients support NAFLD progression [143], whereas dietary habits rich in fruits and vegetables have antioxidant, anti-inflammatory effects, and can improve IR [144]. Protein diets in subjects with T2DM and NAFLD promotes loss of hepatic fat associated with better IR and decreased hepatic cytolytic profile [145], and can improve the glycated haemoglobin A1c (HbA1c) levels [146]. Contrary, others showed that a high protein diet may have negative effects on insulin sensitivity, and its beneficial effects are linked to the amount and quality of the products [147]. Additionally, fructose is commonly added into artificially-sweetened beverages and other sweet solid products. High fructose intake is associated with increased risk of steatosis, liver fibrosis, obesity, and IR [148]. Long-term sucrose ingestion led to increased fat accumulation, glucose intolerance and hyperinsulinemia, and histological damage like increased hepatocyte size and ballooning [149]. Dietary intake of monosaturated FAs found in foods like olive oil and avocado has been shown to improve insulin sensitivity and hepatic fat in prediabetic [150] and in paediatric patients with NAFLD [151]. Those who followed a Mediterranean diet showed a better decrease of liver transaminase, body mass index (BMI) changes, and improvement in IR [152, 153]. Enormous scientific data shows that diet and exercise are first and highly beneficial in metabolic syndromes, and any physician should recommend to their patients additionally to therapeutic medication.

### 4.2. T2DM Medication and NAFLD

The current guidelines endorse that for the HbA1C and serum glycaemic control in T2DM, patients should use one of the seven drug classes approved by the American Diabetes Association (ADA). These classes include metformin, sulfonylureas, thiazolidinediones, dipeptidyl peptidase 4 inhibitors, glucagon-like peptide-1 receptor agonists, sodium-glucose cotransporter 2 inhibitors, and insulin [154]. Substantial research has been made over the last years in order to find among the existing antidiabetic drugs other potential tools that can be used in T2DM-NAFLD patients [155].

#### 4.2.1. Metformin

Metformin also known as Glucophage is the first-line medication for the treatment of type 2 diabetes [156]. This drug lowers both basal and postprandial plasma glucose levels by suppressing the liver gluconeogenesis via phosphorylation of cAMP-response element-binding protein (CREBBP or CBP), decreases intestinal absorption of glucose, and improves insulin sensitivity by increasing peripheral glucose uptake and utilization [157]. It was thought that it could be beneficial along with hypocaloric diet and weight control in nondiabetic patients with NAFLD. However, in many clinical trials that included nondiabetic and diabetic patients, even if metformin administration improved at some degree the serum aminotransferase levels, histological outcomes on long-term failed to show significant differences [158–160]. Nonetheless, metformin has proven to impediment the risk of HCC development and cardiovascular complications related to NAFLD and T2DM [161].

#### 4.2.2. Thiazolidinediones

Thiazolidinediones bind to a transcription factor identified as PPAR-*γ* that enhances the transcription of various genes in the adipose tissue; they induce preadipocyte differentiation into adipocytes, raise adiponectin levels, and help with insulin sensitivity [162]. In a 3-year clinical trial, patients with NAFLD who received Rosiglitazone had reduced liver enzymes and better insulin sensitivity after 1 year of treatment [163]. However, the two-year period following the FILTR-2 extension trial revealed no further improvements or changes in the fibrosis or liver ballooning [164]. Interestingly, a 13 years cross-sectional retrospective analysis noted that pioglitazone use in US patients decreased significantly over the years; however, NAFLD prevalence and incidence increased in T2DM patients which can imply that pioglitazone could have ameliorated this raise [165]. Using long term treatment with pioglitazone along with a hypocaloric diet in prediabetic and T2DM subjects with NAFLD resulted in histological and circulating liver enzymes improvement which persisted over 3 years [166]. Others showed that this drug can improve histologic features but not the mean fibrosis score [167]. In experimental NASH-induced models, pioglitazone administration reduced ceramides and DAG levels and improved the hepatic mitochondria function [168]. These results suggest that this therapy is effective and quite feasible for NAFLD [169]. Currently, there is the ongoing randomized open-label pilot (ToPiND) study that tries to investigate the effects of tofogliflozin 20 mg/day or/and pioglitazone 15–30 mg/day administration. At 6 months and at 1 year of therapy, the hepatic steatosis grade will be measured by MRI-PDFF and collective serum data would be assessed [170]. It remains to be seen whether monotherapy or combination therapy would have the most beneficial effects in NAFLD-T2DM. Many other robust studies are desired however, authors should take into consideration that pioglitazone has many serious side effects such as weight gain, worsening heart failure, osteoporosis, and raised risk of bladder cancer [171].

#### 4.2.3. Glucagon-Like Peptide-1 (GLP-1) Analogues

GLP-1 analogue can promote glucose-mediated insulin secretion, decrease glucagon synthesis, and suppress appetite, which is why it can be a potential medication for NAFLD. Liraglutide administration in NASH subjects resulted in raised insulin sensitivity, decrease of DNL, reduced BMI, cholesterol-LDL, and suppression of lipolysis especially within the subcutaneous adipose tissue [172, 173]. An experimental in vivo and vitro study noted that Liraglutide administration promoted expression of autophagy markers via the AMPK/mTOR pathways leading to antilipotoxic effects [174]. Its ingestion for half a year reduces the subcutaneous body fat (from 361 ± 142 cm^2^ to 339 ± 131 cm^2^) but not visceral, hepatic, or epicardial fat [175]. Also, an open-label, active-controlled parallel-group, multicentre trial showed that liraglutide and sitagliptin added to metformin but not insulin glargine reduced body weight, visceral adipose tissue, and intrahepatic lipid levels in individuals with T2DM and NAFLD [176]. Evidence from clinical trials presents the quality of GLP-1 analogues to become disease-modifying tools in NAFLD [177–179]. The glucose-dependent insulinotropic polypeptide (GIP)/GLP-1 agonist could be used not only for glucose metabolism control but also for NAFLD treatment, as this combination has synergic effects, promotes lipogenesis and weight loss [180]. The physiological effects, the therapeutic implication of GIP antagonism, and agonism in T2DM-NAFLD patients need further exploration in larger human trials.

#### 4.2.4. Sodium-Glucose Cotransporter-2 (SGLT2) Inhibitors

The newly T2DM therapy SGLT-2 inhibitors increase glucagon levels, diminish renal reabsorption of glucose, and increases its excretion. SGLT2 inhibitors could benefit the hepatic function because it promotes glucagon secretion, DNL, and urinary caloric losses with subsequently weight loss [181]. The main SGLT2 representants are composed by canagliflozin, dapagliflozin, and empagliflozin, used as second-line treatment in association with metformin as well as third-line treatment. Molecules such as luseogliflozin and tofogliflozin are only approved in Japan, while ipragliflozin was also approved last year in Russia [154]. A recent systematic review described 8 studies that evaluated the role of SGLT-2 inhibitors on NAFLD. The results illustrated that in most of these studies, patients with SGLT-2 therapy had AST and GGT levels decrease, 5 studies noted reduction in hepatic fat, and 2 studies found improvement in liver fibrosis [182]. Many trials involving canagliflozin [183–186] noted comprehensive results regarding SGLT-2 inhibitors in NAFLD. Also, empagliflozin [187, 188] and dapagliflozin [189, 190] administration showed similar beneficial results. A combination of exenatide once weekly plus dapagliflozin once a day reduced biomarkers of liver steatosis and fibrosis in patients with T2DM uncontrolled by metformin monotherapy [191]. The DURATION-8 (NCT02229396) phase 3 trial displayed similar effects when exenatide once weekly plus dapagliflozin once daily, improved glycemic control and body weight [192–194]. Also, long term use of luseogliflozins led to the improvement of steatosis, fibrosis, and histological activity score [195–197]. Recently, authors demonstrated that the use of the novel SGLT2 inhibitor, NGI001 in high fat diet-induced mice instigates suppression of lipid accumulation, inflammation, upregulation of *β*-oxidation, and they suggest that this inhibitor may be as a new therapeutic approach that can delay the onset of NAFLD [198]. Robust evidence supports the idea that antidiabetic drugs are quite feasible for NAFLD treatment (Table 1), and so far, pioglitazone and liraglutide noted the most promising results. We await further larger clinical trials that can prove better histological outcomes in T2DM-NAFLD patients.

### 4.3. New Potential Therapeutic Targets

New promising treatment options emerged over the years to combat NAFLD [199]. Interestingly, micronutrients like choline and polyphenols also interfere with the liver-gut axis and contribute to several pathways that are crucial for diabetes or the NAFLD development. For example, low choline intake has been associated with worsening liver fibrosis and a higher risk of NAFLD development [200]. The polyphenol family encompasses a wide spectrum of molecules such as curcumin, resveratrol, or quercetin that can be found in vegetables, fruit, and coffee. Recently, researches showed that polyphenol can reduce TG accumulation through antioxidant, and anti-inflammatory effects, and by blockage of lipogenesis via SREBP1c downregulation [201]. Resveratrol administration can regulate PPAR expression, IL-1*β*, and TNF-*α*, and have antisteatosis effects with secondary increase of body weight [202]. Histopathological improvement in NASH models was observed after the administration of a dual PPAR*α*/*γ* agonist named Saroglitazar [203]. Another dual agonist named Elafibranor (GFT505) had similar results in murine models of NAFLD and NASH [204]. (PEGylated) FGF21 analogue marked as Pegbelfermin is also under investigation to see if it can be a feasible tool for NAFLD/NASH treatment [205]. A new potential target that may be used in T2DM treatment is the protein tyrosine phosphatase 1B (PTPIB) that was shown to inhibit insulin signaling and can normalize plasma glucose levels [206]. Considering the rise incidence of NAFLD in men and postmenopausal woman, authors demonstrated in vitro and in vivo study that 17*β*-estradiol (E2) therapy improves IR and fatty acid accumulation by interfering with the JNK activation pathway [207]. Recently, in an open-label prospective studies and trials administration of GS-0976 (Firsocostat), a small molecule inhibitor of acetyl-CoA carboxylase in NASH patients reduced de novo lipogenesis, intrahepatic TG levels, markers of liver injury and steatosis, and may soon be approved by FDA for NASH treatment [208, 209]. Another approach is the inhibition of certain inflammatory pathways involved in NAFLD. Animal studies showed that the inhibition of CCR2 or the ligand CCL2-IR with cenicriviroc may diminish fibrosis especially after 1-year treatment [210]. In a phase 2 randomized, placebo-controlled trial, phase II study, the use of Hepatic thyroid hormone receptor beta (THR-*β*) agonist VK2809 elicited dose dependent improvement on liver fat reduction and liver function [211]. Similar results were obtained when using a similar agent THR-*β* agonist named MGL-3196. However, more studies are needed to mark the side effects of these molecules and their interaction with metabolic systems [212]. A number of drugs that target steatosis and/or fibrosis are currently investigated in Phase II and Phase III clinical trials [213]. More than that, potential therapies that target the gut-liver axis could represent in the future a key strategy in the management of TD2M and NAFLD [214]. For example, the administration of probiotic and synbiotics may reduce histologically-confirmed liver fat and improve liver function [215, 216]. There are many therapeutic possibilities for NAFLD discovered by research teams that we did not discuss here. From the inflammatory, immune, metabolic, oxidative stress, hormonal, and gut-axis pathways to other T2DM therapeutic ways, NAFLD onset and progression should be modulated not just by one single therapeutic approach. Further trials and investigations in this matter are needed.

## 5. Conclusions

For many years, robust evidence tried to demonstrate the association between NAFLD, TD2M, IR, obesity, and other metabolic syndromes. Unfortunately, this is a vast and complex territory and so far, there is no clear evidence that links the glucose metabolism and IR with diabetes and NAFLD manifestations. Many countries lack exhaustive public health response to NAFLD, screening for NAFLD in diabetes patients, awareness campaigns, early validation of risk factors, large scale educational programs, guidelines/algorithms, and long-term strategies for these patients. With the high chance that a steatosis liver advances to NASH especially when multiple comorbidities are associated, early assessment and management of NAFLD in T2DM subjects are imperative. Therapeutic inertia continues to remain a general problem in diabetes; that is why it is crucial that a physician early recognises patients at risk. Clinicians must know what are the best noninvasive tools and therapeutic approaches to prevent and delay NAFLD, and also how to maintain an open collaboration with other specialities. Currently, there is no proper approved pharmaceutical treatment for NAFLD. Multitarget agents or combination of agents should have twice the beneficial effect compared to monotherapy. Numerous trials are currently under development that investigate new promising pharmacological agents for NAFLD and test the effect of antidiabetic drugs on liver function. We hope that in the future, larger clinical trials can assess and approve new therapeutic drugs for NAFLD that can be used safely in T2DM patients.

## Figures and Tables

**Table 1:** Clinical trials which encompass the antidiabetic drug effects on NAFLD. BMI: body mass index; AST: aspartataminotransferaza, ALT: alanine aminotransferase, HbA1c: glycated haemoglobin, DNL: de novo lipogenesis.

Agents	Study type	Dose	Time	Outcomes	Ref.
Rosiglitazone	Randomized placebo-controlled (FLIRT) Trial	4 mg/day 1 month and after 8 mg/day	1 year and 4 months	Improvement of steatosis correlated with reduction of transaminase level improvement in insulin sensitivity.	[163]
	Randomized placebo-controlled (FLIRT 2) extension trial	4 mg/day 1 month and after 8 mg/day	3 years	Improvement in ALT and liver steatosis but no results on ballooning and fibrosis.	[164]
Pioglitazone	Randomized, double-blind, placebo-controlled trial	30 mg/day	2 years	Improvement in individual histologic scores, adipose tissue, hepatic, and muscle insulin sensitivity.	[166]
Pioglitazone vs. ipragliflozin	Open-label, randomized, active-controlled trial	15-30 mg/day vs. 50 mg/day	6 months	AST and ALT, HbA1c, and fasting plasma glucose were similarly reduced in the two-treatment group.	[169]
Liraglutide	Randomised, placebo-controlled phase 2 study (LEAN) study	1.8 mg/day	1 year	Improvements in histological steatosis and hepatocyte ballooning.	[172]
	Double-blind, randomised, placebo-controlled trial	1.8 mg/day	3 months	Reduced BMI, DNL, IR, and hepatic steatosis.	[173]
	Open-label, active-controlled parallel-group, multicentre trial	3 mg/day	6 months	Reduced BMI, hepatic steatosis, and hepatocellular apoptosis.	[176]
	Prospective, single-center study (LEAN-J).	1.2 mg/day	2 years	31% reduction of hepatic steatosis	[179]
Canagliflozin	A prospective study	100 mg/day	6 months	Improvement in histopathologic features and markers of liver dysfunction	[183]
	A prospective study	100 mg/day	6 months	Histological improvement, improvement of IR.	[184]
	Prospective study	100 mg/day	6 months	Improvement in ALT and liver steatosis	[186]
Empagliflozin	Randomised controlled trials (E-LIFT Trial)	10 mg/day	5 months	Improvement in liver steatosis and serum ALT level.	[187]
	Single-arm, open-label, pilot study	25 mg/day	6 months	Reduction in BMI, cholesterol, GGT, ballooning, and fibrosis.	[188]
Dapagliflozin	Randomised controlled trial	10 mg/day	4 months	Reduction in BMI, AST, ALT, and liver steatosis.	[189]
	An open-label, randomized trial	5 mg/day	6 months	Improvements in hepatic steatosis, along with attenuation of fibrosis in a subset of patients with significant fibrosis.	[190]
Luseogliflozin	A prospective, single-arm trial (LEAD trial)	2.5 mg/day	6 months	Improvement of steatosis correlated with reduction of transaminase level	[195]
